# Whole-Genome Sequencing and Analysis Reveals Plant Growth-Promoting Properties and Biocontrol Potential of the *Crotalaria retusa* Endophytic *Bacillus velezensis* Strain G2T39

**DOI:** 10.3390/microorganisms14010123

**Published:** 2026-01-07

**Authors:** Evrad Sausthène Seka Ahoty, Zaka Ghislaine Claude Kouadjo-Zézé, Romain Kouakou Fossou, Anicet Théodore Ebou Ediman, Espérance Pierre-Marie Kéran Boga, Adolphe Zézé

**Affiliations:** 1 Laboratoire de Microbiologie, Biotechnologies et Bio-informatique (LaMBB), UMRI Sciences Agronomiques et Procédés de Transformation, Institut National Polytechnique Félix Houphouët-Boigny, Yamoussoukro 1093, Côte d’Ivoire; evrad.ahoty@inphb.ci (E.S.S.A.); romain.fossou@inphb.ci (R.K.F.); ediman.ebou@inphb.ci (A.T.E.E.); keranboga@gmail.com (E.P.-M.K.B.); 2 Laboratoire Central de Biotechnologies, Centre National de la Recherche Agronomique, Abidjan 1740, Côte d’Ivoire; claudghis@gmail.com

**Keywords:** *Crotalaria retusa*, endophytic *Bacillus*, plant growth-promoting properties, pathogen biocontrol, medicinal plant

## Abstract

*Bacillus velezensis* strain G2T39 is an endophytic bacterium previously isolated from *Crotalaria retusa* L., with evidenced biocontrol activity against *Fusarium oxysporum* f. sp. *Cubense* and *Fusarium graminearum*. In this study, it was shown that this strain also exhibited biocontrol activity against *Colletotrichum gloeosporioides* and *Fusarium oxysporum* f. sp. Vasinfectum, two important crop pathogens in tropical zones. Comprehensive phylogenetic and genomic analyses were performed to further characterize this strain. The genome of *B. velezensis* G2T39 consists of a single circular chromosome of 4,040,830 base pairs, with an average guanine–cytosine (GC) content of 46.35%. Both whole-genome-based phylogeny and average nucleotide identity (ANI) confirmed its identity as *B. velezensis*, being closely related to biocontrol and plant growth promotion Gram-positive model strains such as *B. velezensis* FZB42. Whole-genome annotation revealed 216 carbohydrate-active enzymes and 14 gene clusters responsible for secondary metabolite production, including surfactin, macrolactin, bacillaene, fengycin, bacillibactin, bacilysin, and difficidin. Genes involved in plant defense mechanisms were also identified. Additionally, G2T39 genome harbors multiple plant growth-promoting traits, such as genes associated with nitrogen metabolism (*nifU*, *nifS*, *nifB*, *fixB*, *glnK*) and a putative phosphate metabolism system (*phyC*, *pst glpQA*, *ugpB*, *ugpC*). Additional genes linked to biofilm formation, zinc solubilization, stress tolerance, siderophore production and regulation, nitrate reduction, riboflavin and nicotinamide synthesis, lactate metabolism, and homeostasis of potassium and magnesium were also identified. These findings highlight the genetic basis underlying the biocontrol capacity and plant growth-promoting properties of *B. velezensis* G2T39 and support its potential application as a sustainable bioinoculant in agriculture.

## 1. Introduction

Global agricultural productivity plays a pivotal role in ensuring food security for an ever-growing population. However, plant diseases caused by pathogenic microorganisms continue to significantly threaten crop yields worldwide, undermining agricultural sustainability and food systems [1]. To counter these losses, the extensive use of chemical pesticides has become a common practice in modern farming [2]. While effective, the excessive and long-term application of agrochemicals has raised serious environmental concerns, including soil degradation, ecosystem contamination, and the emergence of resistant pathogen strains. Considering these challenges, there is a growing global demand for sustainable and environmentally friendly plant disease management strategies. Many countries are actively promoting alternative biosolutions, such as green pesticides and microbial biocontrol agents [3,4]. Among these, plant growth-promoting microorganisms (PGPMs), notably bacteria belonging to the genera *Pseudomonas*, *Bacillus*, and *Streptomyces*, have gained increasing attention due to their multifaceted benefits. These microorganisms not only enhance plant growth and stress tolerance but also contribute to the suppression of phytopathogens [5,6,7]. Within this group, the genus *Bacillus* stands out owing to its genetic diversity, ease of cultivation, and spore-forming ability, which confers long-term stability and commercial viability. Several *Bacillus* species have demonstrated significant potential as biofertilizers and biopesticides, supporting their application in sustainable agriculture [8]. In particular, *Bacillus velezensis*, a reclassified species related to *B. amyloliquefaciens* [9], has drawn attention as a key PGPM. Strains of *B. velezensis* are known to produce a diverse array of antimicrobial metabolites, including lipopeptides (surfactin, bacillomycin D, fengycin, bacillibactin) and polyketides (macrolactin, bacillaene, difficidin), many of which contribute directly to the inhibition of plant pathogens [10]. For instance, *B. velezensis* FZB42 allocates approximately 8.5% of its genome to the biosynthesis of antibiotics and siderophores via nonribosomal peptide synthetase (NRPS) and polyketide synthase (PKS) pathways [11]. Specific compounds such as fengycin and bacillomycin D have shown synergistic antifungal activity against *Fusarium oxysporum* [12], while iturin and fengycin produced by *B. amyloliquefaciens* strains CECT 8237 and CECT 8238 display broad-spectrum antimicrobial effects [13]. Furthermore, genomic studies have revealed that *B. velezensis* strains isolated from a variety of hosts exhibit both plant growth-promoting and disease-suppressing traits [14,15,16,17,18]. In this trend, endophytic *Bacillus* species can be considered as a multifaceted toolbox for agriculture, environment, and medicine. Indeed, endophytic *B. velenzensis* strains with similar bioactivities have been isolated from medicinal and woody plants, including *Cinnamomum camphora* [19,20]. The recent isolation of various endophytic bacteria, including strain G2T39 from the medicinal plant *C. retusa,* followed by small-scale phenotypic analyses, demonstrated notable biocontrol potential against *Fusarium graminearum* and *Fusarium oxysporum* f. sp. *Cubense* isolated from temperate regions [21]. Despite these advances, genomic and phylogenetic data remain limited for many newly identified *B. velezensis* strains. Given the observed antagonistic activity of strain G2T39 against *Fusarium*, we hypothesized that it may possess genomic determinants for antimicrobial production and plant growth promotion. Therefore, the objectives of this study were to (1) perform strain G2T39 whole-genome sequencing and analyses; and (2) predict gene functions that may support its functional characteristics.

## 2. Materials and Methods

### 2.1. Evaluation of B. velezensis Strain G2T39 Antagonistic Activity Against Phypathogenic Agents from Côte d’Ivoire

The antagonistic activity of *Bacillus velezensis* strain G2T39 was assessed in a previous work [21]. Strain G2T39 was obtained from the Laboratoire de Microbiologie, Biotechnologie et Bioinformatique (Institut National Polytechnique Félix Houphouët-Boigny, Côte d’Ivoire) In this study, G2T39 was evaluated against two important phytopathogenic fungi, *Colletotrichum gloeosporioides* and *Fusarium oxysporum* f. sp. *vasinfectum* (FOV), causing diseases on mango and cotton, respectively, in Côte d’Ivoire, using an in vitro dual culture assay as described by Munakata et al., [22]. *C. gloeosporioides* and *F. oxysporum* f. sp. *vasinfectum* (FOV) were obtained from the Laboratoire Central de Biotechnologie (LCB), Centre National de Recherche Agronomique (CNRA), Côte d’Ivoire, and the assay was conducted as described by Ahoty et al. (2025) [21]. Briefly, 5 mm mycelial plugs were excised from the edge of actively growing fungal cultures and placed at the center of 9 cm diameter Petri dishes containing 12 mL of Potato Dextrose Agar (PDA). G2T39 bacterial cultures were grown in 2 mL of Nutrient Broth (NB) at 28 °C for either 24 or 72 h. A 50 µL aliquot of the bacterial suspension was then spotted 3 cm from the fungal plug on the same plate. Plates were incubated at 28 °C for five days or nine days according to the fungal pathogen. Each assay was performed in triplicate. Control plates were inoculated with the fungal pathogen only, without bacterial interference. Fungal growth inhibition was quantified by calculating the inhibition rate (%) [21,23].

### 2.2. Nitrogen Fixation Test

Strain G2T39 nitrogen-fixing capability was assessed using the nitrogen-free medium Burk’s N-Free, prepared with the following composition (per liter): sucrose, 20.0 g; K_2_HPO_4_, 0.64 g; KH_2_PO_4_, 0.16 g; MgSO_4_·7H_2_O, 0.20 g; NaCl, 0.20 g; CaSO_4_·2H_2_O, 0.05 g; and agar, 15 g. Supplementary components, including Na_2_MoO_4_·2H_2_O (0.05%, 5.0 mL) and FeSO_4_·7H_2_O (0.3%, 5.0 mL), were filter-sterilized before being added to the autoclaved medium. The pH was adjusted to 7.3 prior to sterilization (121 °C for 15 min). Strain G2T39 was cultured on this medium at 28 °C for 48 h, following the protocol described by Park et al. [24].

### 2.3. Whole-Genome Sequencing and Assembly of G2T39

The genome of *B. velezensis* strain G2T39 was sequenced using the Pacbio Sequel II and DNBSEQ platforms at the Beijing Genomics Institute (BGI, Shenzhen, China). Four SMRT cell zero-mode waveguide arrays of sequencing were used by the PacBio platform to generate the subread set. PacBio subreads (lengths < 1 kb) were removed. The program Canu was used for self-correction. Draft genomic unitigs, which are uncontested groups of fragments, were assembled using Canu (Canu v2.2 [25] (useGrid = 0 and corOvlMemory = 4) from a high-quality corrected circular consensus sequence subread set. To improve genome accuracy, GATK (https://gatk.broadinstitute.org/hc/en-us (accessed on 7 October 2024)) was used to perform single-base corrections.

### 2.4. Genome Component

Gene prediction was conducted using Glimmer v3.02 [26] based on Hidden Markov Models (-l linear). Functional RNA elements were identified as follows: tRNAs using tRNAscan-SE v1.3.1; rRNAs using RNAmmer; and sRNA using the RFAM database. Tandem repeats were detected using Tandem Repeat Finder (http://tandem.bu.edu/trf/trf.html (accessed on 7 October 2024)), and microsatellites/minisatellites were selected based on repeat unit length and frequency. Genomic islands were predicted using the Genomic Island Suite of Tools (GISTs) (http://www5.esu.edu/cpsc/bioinfo/software/GIST (accessed on 7 October 2024)), incorporating IslandPath-DIMOB, SIGI-HMM, and IslandPicker algorithms.

Functional annotation of predicted proteins was performed using BLAST+2.15.0 against the following seven databases: Clusters of Orthologous Groups (COG), Kyoto Encyclopedia of Genes and Genomes (KEGG), Gene Ontology (GO), Non-Redundant Protein Database databases (NR), UniProtKB (UniProtKB/TrEMBL and UniProtKB/Swiss-Prot), and EggNOG v6.0. Synteny analysis between G2T39 and related strains (*Bacillus* strains FZB42, SQR9, DSM 7, and XJ5) was conducted using MUMmer and BLAST. Core and pan-genomes were identified using CD-HIT with a 50% pairwise identity threshold and a 0.7 length difference cutoff. Gene family clustering involved a multi-step pipeline: (i) protein sequences were aligned using BLAST; (ii) redundant alignments were filtered using Solar; and (iii) gene family clustering was conducted using hcluster_sg. Moreover, the genome sequence data of G2T39 were uploaded to the Type (Strain) Genome Server (TYGS), a free bioinformatics platform available under https://tygs.dsmz.de (accessed on 7 September 2025), for whole-genome-based taxonomic and phylogenetic analysis [27]. Briefly, a phylogenetic tree was constructed using the Genome BLAST Distance Phylogeny (GBDP) approach. Branch lengths were scaled in terms of the GBDP distance formula *d5*, and branch support was inferred from 100 pseudo-bootstrap replicates. Bootstrap values > 60% are shown in the tree [27]. Average nucleotide identity (ANI) values between *B. velezensis* strain G2T39 and *Bacillus* reference strains were calculated with JspeciesWS [28]. Secondary metabolite biosynthetic clusters were identified using AntiSMASH v8.0.2 [29] with default parameters, referencing the MIBiG r4.0 database for structure and classification [30]. In addition, putative genes encoding carbohydrate-active enzymes (CAZymes) were annotated using the dbCAN3 meta server [31], which integrates HMMER (for domain prediction) [32], Hotpep (for short motif detection) [33], and DIAMOND (for similarity search against CAZy) [34]. Identified CAZymes genes were further analyzed using SignalP 5.0 [35] to predict the presence of N-terminal signal peptides indicative of potential secretion pathways.

## 3. Results

### 3.1. Biological Control Activity of B. velezensis Strain G2T39

**Figure 1:**
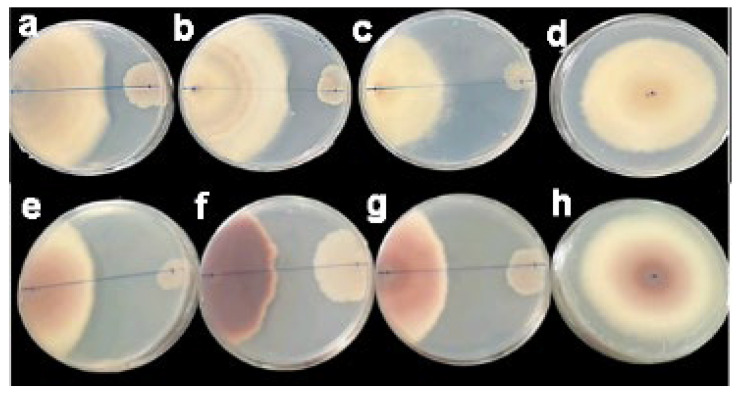
Antimicrobial activity of *B. velezensis* G2T39 against *Colletotrichum gloeosporioides* and *Fusarium oxysporum* f. sp. *vasinfectum.* The assay, performed in triplicate, showed that in the presence of G2T39, the growth of *C. gloeosporioides* (**a**–**c**) and *F. oxysporum* f. sp. *vasinfectum* (**e**–**g**) was impaired. In contrast, the pathogenic fungi grew well without inhibition on the control plates (**d**,**h**).

Here, we evaluated *B. velenzensis* G2T39 antagonistic activity against two fungal phytopathogens, *C. gloeosporioides* and *F. oxysporum* f. sp. *vasinfectum* (FOV). Indeed, in the presence of G2T39, the growth of *C. gloeosporioides* and *F. oxysporum* f. sp. *vasinfectum* (FOV) was impaired (Figure 1), with inhibition rates of 30% and 45%, respectively (Supplementary Table S1).

### 3.2. Whole-Genome Sequencing and Phylogeny of B. velezensis Strain G2T39

**Figure 2:**
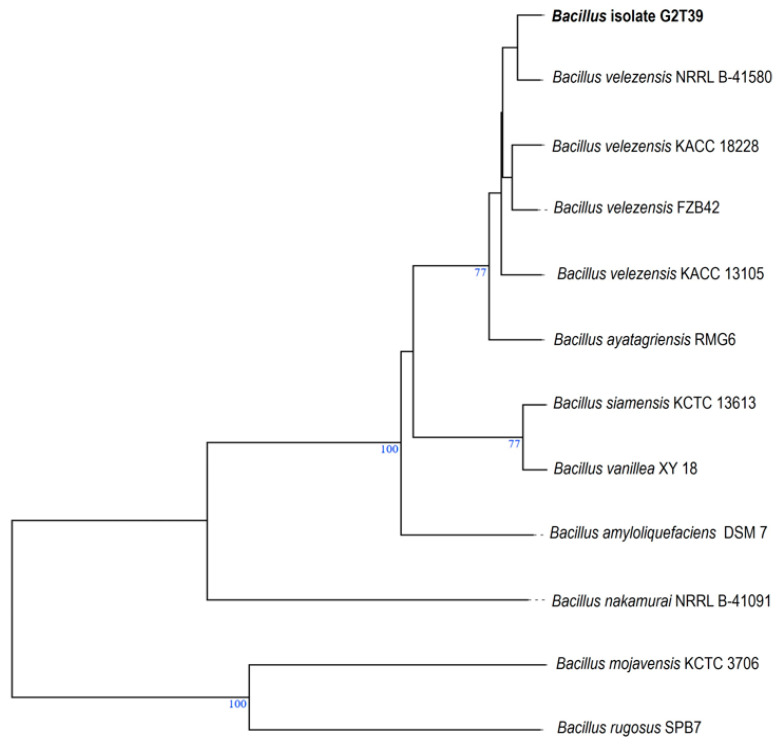
Whole-genome phylogenetic tree built with the TYGS platform. About 10 strains closely related to G2T39 were automatically found by TYGS, and the tree was inferred from GBDP distances calculated from their genome sequences. *Bacillus* isolate G2T39 was shown in bold. Bootstrap data (values > 60%) from 100 replications are shown as a percent for each branch. Branch lengths were scaled in terms of the GBDP distance formula d5. The tree was manually edited and, where necessary, the names of reference strains were updated (e.g., *Bacillus velezensis* FZB42, was coined as *Bacillus amyloliquefaciens* subsp. plantarum FZB42 in the TYGS platform; https://tygs.dsmz.de (accessed on 7 September 2025).

The genome of the endophytic bacteria strain G2T39 was sequenced using Oxford Nanopore technology. Prior to genomic and functional comparisons, we refined the identification and taxonomy of G2T39 using whole-genome-based phylogeny. Refinement of the presumptive affiliation of G2T39 was important to ensure accurate selection of its best reference strains for subsequent analysis, including structural synteny and pairwise average nucleotide identity. In a previous study, BlastN analysis that used only a partial 16S rRNA gene sequence (<1000 bp) was used to identify the strain G2T39 as *B. velezensis* [21]. Here, a genome-based phylogenetic tree produced with the TYGS platform revealed that G2T39 unambiguously belongs to the *B. velezensis* species (Figure 2).

Moreover, average nucleotide identity (ANI) calculations showed that G2T39 and several *Bacillus velezensis* strains shared the highest ANI BLAST (ANIb) and ANI Mummer (ANIm) values of 98.83% and 99.00%, respectively (Table 1). Clearly, both calculated ANIb and ANIm scores were higher than the 95–96% threshold proposed for delineating microbial species [36], confirming that G2T39 belongs to the *B. velezensis* species. Interestingly, G2T39 is also closely related to key Gram-positive *Bacillus* model strains for plant growth promotion and biocontrol, such as *B. velezensis* FZB42 [37] (Table 1; Figure 2).

### 3.3. Genomic Profiling of B. velezensis G2T39

**Figure 3:**
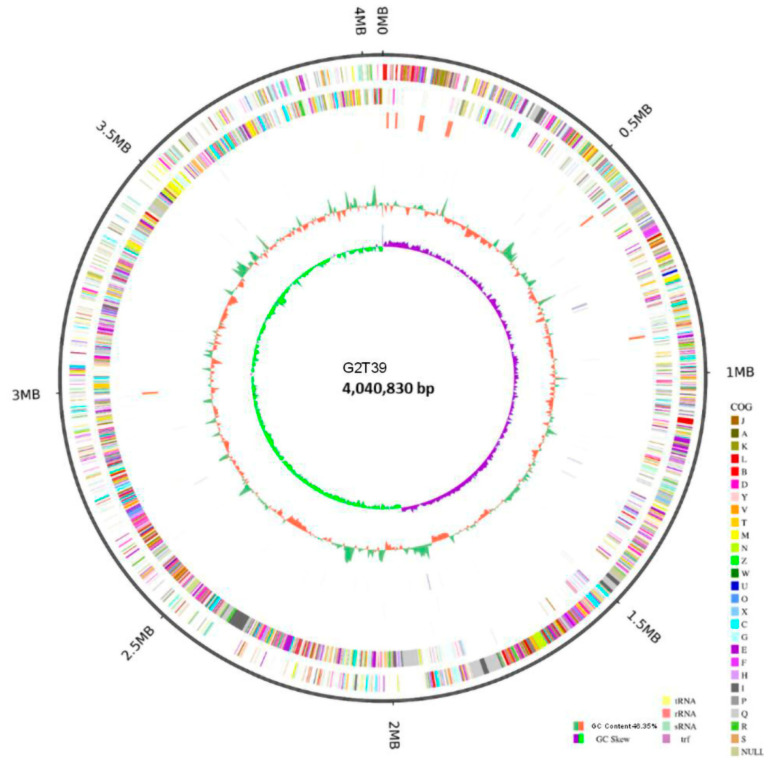
Circular genome map of *Bacillus velezensis* G2T39. From the innermost to the outermost: ring 1 shows GC skew positive (light orange) and negative (blue) values; ring 2 for GC content, where green indicates values greater than the average and red indicates values lower than the average; ring 3 (reverse strand) and ring 4 (forward strand) show the distribution of CDSs (gray), tRNAs (light green), rRNAs (light red), ncRNAs (orange), and ncRNA regions (light blue); ring 5 indicates genome size (black line).

The genome of *B. velezensis* strain G2T39 (accession CP199939) consisted of a single circular chromosome measuring 4,040,830 base pairs (bp), with an average guanine–cytosine (GC) content of 46.35% (Figure 3).

Eleven (11) databases, including VFDB, ARDB, CAZY, IPR, SWISSPROT, COG, CARD, GO, KEGG, NR, and T3SS, were used for gene function annotation of *B. velezensis* strain G2T39 (Table 2). The G2T39 genome comprises 4118 coding genes, including 3911 protein-coding sequences (CDSs), 87 transfer RNAs (tRNAs), 27 ribosomal RNAs (rRNAs), 33 non-coding RNAs (ncRNs), and 4 small open reading frames (sORFs). Of these coding genes, 4090 (99.32%), 3444 (83.63%), 3323 (80.69%), 3030 (73.57%), 2561 (62.19%), and 2387 (57.96%) matched entries in the NR, IPR, SWISSPROT, COG, KEGG, and GO databases, respectively. Thus, four databases showed the highest match values (>70%), namely COG, IPR, NR, and Swiss-Prot, while a second group of databases included GO and KEGG (>50%) (Table 2).

**Figure 4:**
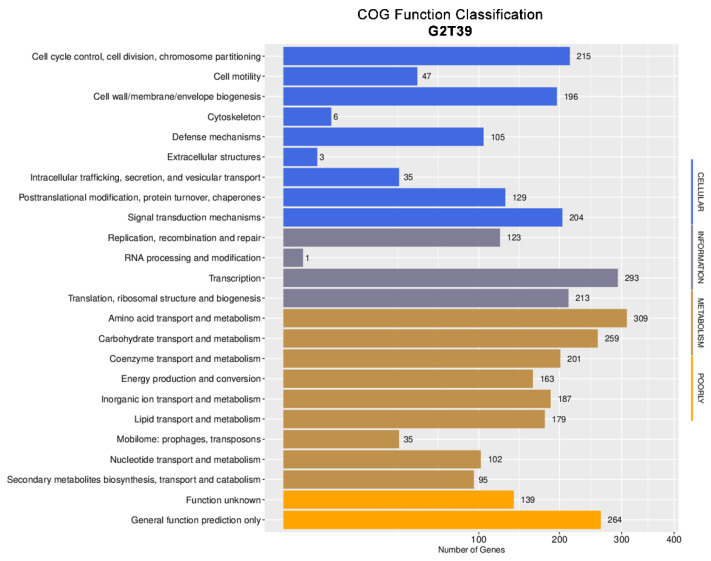
Distribution of genes across COG functional categories in the G2T39 strain genome.

The COG database served as the basis for further analysis in this study. Within the COG database, 3100 genes were assigned to specific functional groups, while 403 were classified as having general function prediction or unknown function (Figure 4).

The metabolism functional group was the most abundant, accounting for 1530 genes (49.35%), reflecting the importance of these functions for this bacterium. Within the metabolism class, the amino acid transport and metabolism category was the most abundant (20.19%), followed by the carbohydrate transport and metabolism category (13.13%).

Concerning KEGG annotation (Figure S1), 2571 genes were assigned to 42 KEGG pathways grouped into six classes, including cellular processes, environmental, genetic, human diseases, metabolism, and organismal systems. This high diversity of pathways, compared with *B. velezensis* bacterium Y89 with biocontrol potential [15], may reflect a possible adaptability of G2T39 to various environments. According to GO annotation, genes were classified into 34 functional groups within three main classes, including biological processes, cellular components, and molecular function (Figure S2). Of these classes, genes involved in biological processes were the most abundant. Within the biological process class, the number of genes related to cellular processes was the highest.

### 3.4. Genomic Comparison and Structural Synteny of B. velezensis G2T39

Genomic comparisons were performed between *Bacillus velezensis* G2T39 and four selected reference strains, namely *B. velezensis* FZB42, *B. velezensis* SQR9, *B. amyloliquefaciens* DSM7, and *B. amyloliquefaciens* XJ5 (Table 3). The reference strains were selected based on phylogenetic relationship and ANI data (Figure 2; Table 1). They included strains closely related to G2T39 and/or key *Bacillus* model strains for plant growth promotion and biocontrol commonly used in genomic comparative analyses (e.g., FZB42 and DSM 7) [3,38]. The five isolates shared similar genomic features (genome size of ca. 4 Mbp and ca. 4000 CDS), although they were isolated from contrasted environments, including soil and the rhizosphere. Analysis of mobile genetic elements and tandem repeats revealed that *Bacillus velezensis* G2T39 possesses a moderately dynamic genome relative to closely related strains. G2T39 harbors four prophage regions, including one intact element, indicating the presence of at least one potentially functional prophage alongside remnants of past phage integrations. This prophage load is comparable to that of *B. amyloliquefaciens* XJ5 but shows higher integrity than that of *B. velezensis* FZB42 and *B. velezensis* SQR9, which contain only incomplete or questionable elements. In contrast, *B. amyloliquefaciens* DSM7 exhibits a markedly more active mobilome, with eight prophage regions, including five intact. The tandem repeat landscape further supports an intermediate genome plasticity in G2T39: although it lacks microsatellites, it contains 36 minisatellites, a number exceeding those of XJ5 and SQR9 and comparable to FZB42, but considerably lower than that of the highly repetitive DSM7. Together, these features suggest that G2T39 maintains moderate genomic flexibility while avoiding the extensive repeat accumulation and prophage activity seen in more dynamic soil-associated strains such as DSM7. The main findings are summarized in Table 3.

**Figure 5:**
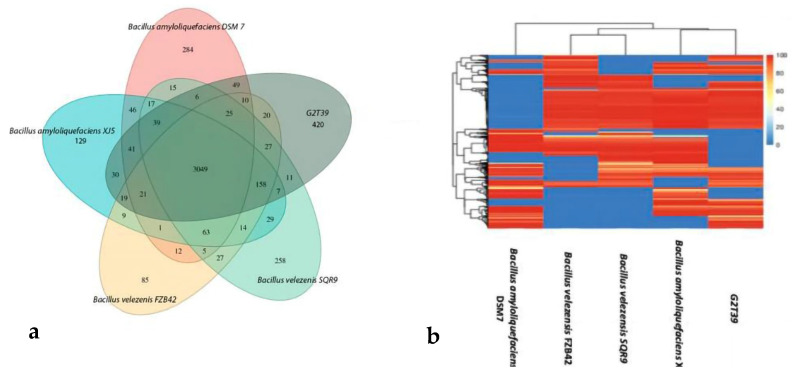
(**a**) Venn diagram showing the number of clusters of orthologous genes shared by G2T39, *B. velezensis* FZB42, *B. velezensis* SQR9, *B. amyloquefaciens* XJ5 and *B. amyloquefaciens* DSM 7, as well as unique genes. (**b**) Heatmap of dispensable genes cluster in G2T39, *B. velezensis* FZB42, *B. velezensis* SQR9, *B. amyloquefaciens* XJ5, and *B. amyloquefaciens* DSM 7. The top panel shows strain clusters, while gene similarity is shown in the middle, with different colors representing different levels of coverage in the heatmap. Color/depth is indicated in the top-right pic.

Moreover, a core- and pan-genome analysis including *B. velezensis* FZB42, *B. velezensis* SQR9, *B. amyloliquefaciens* QSM7, *B. amyloliquefaciens XJ5,* and G2T39 was performed. It was shown that the number of non-redundant pan-genes between G2T39 and the four reference strains was 5916, among which the number of core genes was 3049. The number of non-shared dispensable genes was 1091, of which 420 genes (7.09%), the highest number, were specific to G2T39 (Figure 5a). The heatmap of dispensable genes revealed that G2T39 and *B. amyloliquefaciens XJ5* shared higher numbers, while *B. velezensis* FZB42 and *B. velezensis* SQR9 formed another branch. Moreover, *B. amyloliquefaciens* QSM7 formed a unique branch (Figure 5b). The overlaps among strains indicate a broad conservation of gene content, with differences mainly due to accessory genomic regions. The heatmap displays patterns of presence and absence across orthologous gene clusters, highlighting both conserved gene families and clusters that vary among strains. While the genomes share a large common backbone, distinct blocks of gene families are differentially represented, illustrating functional divergence at the accessory genome level.

**Figure 6:**
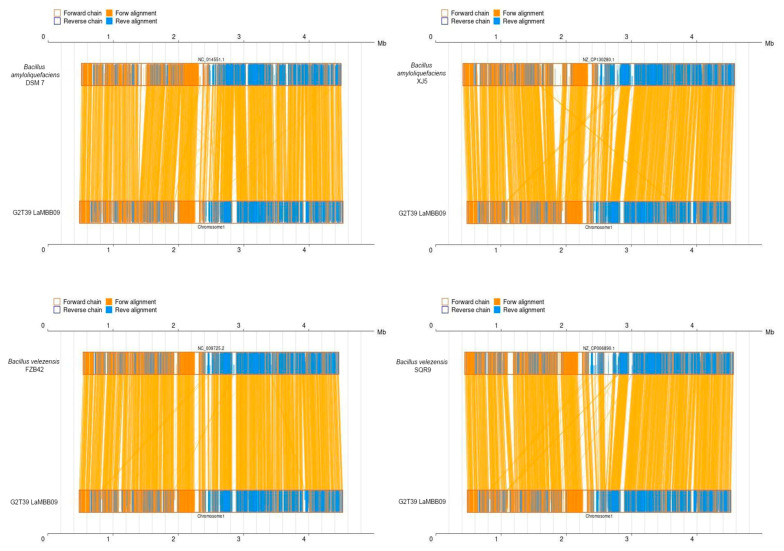
Pairwise synteny of the genome of strain G2T39 with the genomes of other Bacillus (XJ5, DMS7, SQR9, and FZB42) at the amino acid level. Yellow boxes stand for forward chains and blue boxes stand for reverse chains within the upper and following sequence regions. Within the sequence boxes, yellow regions stand for nucleic acid sequences on the forward chain of this genome sequence, while blue regions stand for nucleic acid sequences on the reverse chain of this genome sequence. In the middle region between two sequences, yellow lines stand for forward alignments and blue lines stand for reverse complementary alignments.

Pairwise synteny analysis revealed that *strain G2T39* shares a highly conserved genome architecture with both *B. amyloliquefaciens* and *B. velezensis* strains (Figure 6). All four reference genomes exhibited strong macrosynteny with G2T39, with the central portion of the chromosome (≈0.5–3.8 Mb) showing dense forward alignments and highly conserved gene order. Only minor local inversions were detected within this core region. In contrast, the terminal regions of the chromosome displayed structural variability, including fragmented alignments and small-scale inversions, consistent with the presence of accessory genomic elements such as mobile elements and biosynthetic gene clusters. Among the reference strains, *B. velezensis* FZB42 and SQR9 showed the highest structural similarity to G2T39, whereas *B. amyloliquefaciens* DSM 7 and XJ5 exhibited slightly more localized rearrangements. No major chromosomal inversions, translocations, or large-scale rearrangements were observed in any comparison.

### 3.5. Prediction of G2T39 Genome-Wide Secondary Metabolites and CAZymes

AntiSMASH v8.0.2 analysis identified 14 putative secondary metabolite biosynthetic gene clusters in the G2T39 genome, supporting its potential for antimicrobial compound production (Table 4).

**Figure 7:**
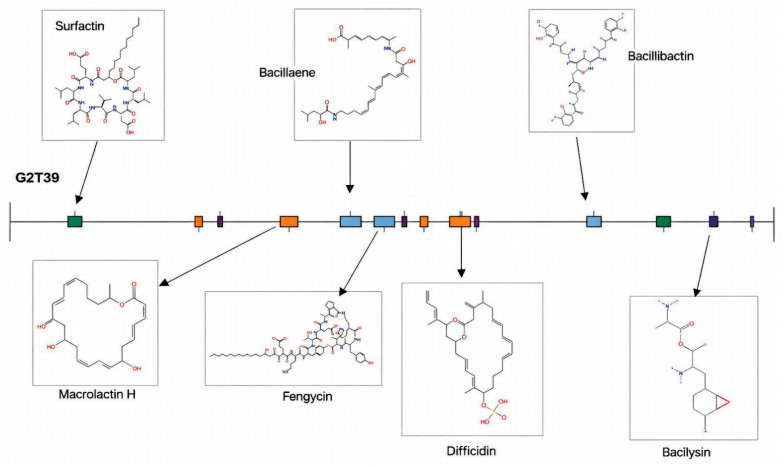
Biosynthetic gene clusters identified in the *B. velezensis* G2T39 genome. Green (Non-Ribosomal Peptide Synthesis Pathways), Orange (Polyketide synthases), Purple (Terpenes), Light blue (Hybrids), Blue (Ribosomally synthesized and post-translationally modified peptides) and Dark blue (others).

Fourteen biosynthetic gene clusters (BGCs) were identified, seven of which were annotated with high confidence based on sequence similarity to characterized clusters (Figure 7). The first cluster encodes a nonribosomal peptide synthetase (NRPS) highly similar to the surfactin-producing cluster previously reported in *Bacillus velezensis* FZB42 (BGC0000433.5). The second cluster corresponds to a polyketide synthase (PKS) with homology to the macrolactin H cluster of FZB42 and *Jeotgalibacillus marinus* (BGC0001383.3), which is also associated with the production of macrolactin B, macrolatin 1C, and macrolactin E. The third cluster is a hybrid NRPS-PKS type, showing high similarity to the bacillaene BGC of FZB42. The fourth is an NRPS-betalactone type cluster related to fengycin biosynthesis, while the fifth is associated with difficidin production and shares similarity with the PKS cluster of FZB42. The sixth cluster encodes an NRPS involved in bacillibactin synthesis (BGC0001185.8), and the seventh corresponds to the bacilysin biosynthetic gene cluster (BGC0001184.4) from FZB42. The remaining seven clusters displayed low similarity to known BGCs and were therefore considered putative. These include an NRPS cluster resembling the choline-type BGC of *Aspergillus nidulans* (BGC0002276.2), a terpene cluster associated with 4-hydroxy-3-nitrosobenzamide production from *Streptomyces murayamaensis* (BGC0000885.3), and another terpene cluster potentially involved in the biosynthesis of fumihopaside A and related compounds with anti-stress properties in *Aspergillus fumigatus*. Moreover, a PKS cluster was identified with similarity to that of *Streptomyces griseus* subsp. *griseus* NBRC 13350, which is known to produce 2-methoxy-5-methyl-6-(13-methyltetradecyl)-1,4-benzoquinone and related phenolic compounds. The G2T39 genome displays a notable genetic basis for anti-pathogen activity. Indeed, it encodes active compounds such as bacillaeane and difficidin, which are antibacterial compounds. Moreover, it also encodes antifungal compounds like surfactin and fengycin. Additionally, some dual antibacterial–antifungal compounds, such as macrolactin and bacilysin, were found in the genome. Notably, a gene cluster found in the G2T39 genome encodes a siderophore product (bacillibactin), which impedes the growth of bacterial and fungal competitors of phytopathogens by competing for essential ions and thus plays a significant role in expediting the procurement of ferric ions from minerals and rhizosphere compounds.

Moreover, the *B. velezensis* G2T39 strain genome contained 216 CAZyme genes grouped into 68 classes (Supplementary Table S2). Three had auxiliary activity, forty-nine were carbohydrate-binding modules (CBMs), fifteen carbohydrate esterases, eighty-two glycoside hydrolases (GHs), sixty-four glycosyl transferases (GTs), and three polysaccharide lyase genes. Glycoside hydrolases and glycosyl transferases were shown to be abundant in the G2T39 genome compared to the closely related *B. velezensis* strains SQR9 and FZB42 (Table 5). Of the 49 CBMs, the most abundant in the G2T39 genome was the CBM50 class that contained 34 members, of which 12 genes were homologous (99% at least) to CBM50 belonging to various *Bacillus* species (Supplementary Table S3). The 82 were grouped into 32 families of which GH13 was the most abundant with six genes highly homologous (99–100%) to GH13 belonging to diverse *Bacillus amyloliquefaciens* and *B. velezensis*. Additionally, GH1, GH43, GH30, GH51, GH73, GH46, GH32, GH18, GH13, GH0, GH1, GH126, GH23, and GH4 were highly homologous (99–100%) to corresponding families in diverse *Bacillus* species (Supplementary Table S1). Of the 64 glycosyl transferases, GT2 was the most abundant in the G2T39 genome, with 25 genes, of which 13 were highly homologous (99–100%) to GT2 belonging to different *Bacillus*, including *B. velezensis* and *B. amyloliquefaciens*. The G2T39 genome also contains eight GT4 genes, five GT51 genes, two GT1 genes, one GT0 gene, and one GT8 gene, all of which were highly homologous to those belonging to different *Bacillus* species.

### 3.6. Genetic Basis of G2T39 Plant Defense Activity

The G2T39 genome possessed genes involved in pathogen-associated molecular pattern-triggered immunity (PTI) and induced system resistance (ISR) (Table 6). Four genes, including *tuf*, *efp*, and *tsf*, encoding translation elongation factors, and *dacA*, encoding D-alanyl-D-alanine carboxypeptidase, are involved in PTI. Moreover, four genes, including *ilvB* and *alsS*, associated with acetolactate synthase, *metC*, encoding cystathionine beta-lyase, and *luxS*, encoding S-ribosylhomocysteine lyase, are involved in ISR.

### 3.7. Genetic Basis of Genes Associated with Nitrogen Metabolism/Fe–S Cluster Assembly and Nitrogen Regulation in G2T39 Genome

G2T39 could grow on Burk’s medium after 48 h of culture, showing a presumptive nitrogen-fixing capacity (Supplementary Figure S3). Moreover, genome sequencing and analysis showed that the G2T39 genome harbored genes directly involved in the biosynthesis of the nitrogenase, including an *nifS* gene cluster encoding for a cysteine desulfurase, *skfB* encoding a putative Fe-S oxydoreductase, *sufB* encoding a Fe-S cluster assembly scaffold protein, *iscU*, *nifU* encoding a Fe-S cluster assembly scaffold protein, and the gene *iscA* encoding an Fe-S cluster assembly iron-binding protein. Additionally, the G2T39 genome exhibited two nitrogenase regulatory genes, including *fixB*, which encodes an electron transfer flavoprotein, an enzyme involved in nitrogen fixation, the gene *glnK*, encoding the nitrogenase regulatory protein PII, and the gene *degU*, coding for a two-component regulator of nitrogenase (Table 7).

### 3.8. Genetic Basis for Plant Growth-Promoting Activity of G2T39

The *B. velezensis* G2T39 genome exhibited different genes encoding proteins predicted to be associated with multiple plant growth-promoting functions, including nitrogen metabolism, phosphate metabolism, biofilm formation, siderophore production, riboflavin synthesis, zinc solubilization, stress tolerance, nitrate reduction, lactate metabolism, potassium homeostasis, magnesium transport, and cytochrome c biogenesis. Despite the presence of multiple genes encoding growth-promoting functions in the G2T39 genome, only genes 80% homologous to databases were selected. Indeed, the genome of *B. velenzensis* strain G2T39 harbored different genes involved in phosphate metabolism, including *glpQA*, encoding a glycerophosphoryl diester phosphodiesterase, *phyC*, encoding a phytase, and the genes *ugpE*, *ugpB*, and *ugpA* (Table 8).

The G2T39 genome harbored various genes involved in different plant growth functions, as shown in Table 9.

## 4. Discussion

Since endophytic *Bacillus* can be considered an important multifaceted toolbox for agriculture [73], better characterization of the strain G2T39 appeared to be urgent. A phylogenomic analysis revealed that *B. velezensis* strain G2T39 exhibited a high degree of homology with *B. velezensis* NRRL B-41580, the type strain for *B. velezensis* bacterium [74]. Moreover, strain G2T39 was also closely related to key Gram-positive *Bacillus* model strains for plant growth promotion and biocontrol, such as *B. velezensis* FZB42 [37] and *B. velezensis* SQR9. These data were further supported by average nucleotide identity (ANI) analysis. *B. velezensis* SQR9 was isolated from the cucumber rhizosphere, while FZB42 was isolated from the beet rhizosphere [75]. The close relatedness between these rhizospheric *Bacillus* strains and G2T39 isolated from *C. retusa* stems, has no significant correlation with their origin.

Besides its capacity to control *Fusarium* from temperate zones, as already evidenced [21], G2T39 could also control phytopatogens such as *C. gloeosporioides* and *F. oxysporum* f. sp. *vasinfectum* from tropical zones, showing its broad-spectrum biocontrol potential. Biological control potential has also been reported for *B. velenzensis* strain BN [18] and its closely related *Bacillus* strains such as SQR9 [76] and FZB42 [77]. Moreover, various *Bacillus* isolates close to *B. subtilis*, *B. atrophaeus*, *B. amyloliquefaciens*, *B. cereus*, *B. licheniformis*, and *B. pumilus* have been described as biocontrol agents against different *Fusarium* species, [20,22,77,78,79,80] sometimes exhibiting broad-spectrum activity, as evidenced with *B. velezensis* BN (18).

This antagonistic charateristic of G2T39 could be due to the presence in its genome of nonribosomal peptide synthetase (NRPS), macrolactin, fengycin, difficidin, bacillibactin, and bacilysin, all of which are antibacterial compounds. Additionally, it also encodes antifungal compounds like surfactin and fengycin. Inhibitory activity of *B. velezensis* and other *Bacillus* can be attributed to several key factors related to their biological and biochemical properties, such as production of antimicrobial compounds, siderophore production, and the production of hydrolytic enzymes [77]. Indeed, the *Bacillus* genus is known to produce a huge diversity of antibacterial compounds [81,82]. Moreover, genome sequencing and analysis have recently revealed that the ability of *B. velezensis* to function as a biocontrol bacterium is mainly due to the fact that this species produces various secondary metabolites and enzymes [14,18,83]. Indeed, in general, *B. velezensis* species produces secondary metabolites such as antimicrobial cyclic lipopeptides synthesized by NRPS and polyketides synthesized by polyketide synthases, which are very important for their broad-spectrum antimicrobial activity [15,18]. The G2T39 genome harbored an NRPS closely related to the surfactin-producing cluster previously reported in *B. velezensis* FZB42 [84].

Moreover, antifungal activities from microbes can also be mediated by enzymes able to hydrolyze fungi chitin [85]. The most well-known enzymes associated with such functions are CAZymes, which cleave polysaccharides and other structural compounds and thus allow antifungal activities in plant growth-promoting bacteria such as *Bacillus* [15]. CAZyme prediction of the endophytic strain *B. velezensis* G2T39 suggested that this strain might play a significant role in antifungal production and activity. Indeed, the G2T39 strain harbors the CBM50 family, which contains 34 members known to promote antifungal activity by binding to the chitinous component of the fungal cell wall, as already evidenced [86,87]. Of diverse GHs, the G2T39 genome harbored GH18, GH19, GH23, and GH73, which can act as chitinases or peptidoglycanases, conferring antibacterial and antifungal properties simultaneously. CAZymes were shown to play an important role in spatial competition, nutrient acquisition, and suppression of the sugarcane red rot pathogen in YC89, an endophytic *B. velezensis* [15].

It is also known that the biocontrol effects exerted by antagonistically acting *B. velezensis* can be due to the stimulation or induction of plant resistance through ISR, PTI, and ETI [15,84]. It was shown that the G2T39 genome harbored genes such as *tuf*, *efp*, and *tsf*, encoding translation elongation factors that are involved in pathogen-associated molecular pattern-triggered immunity (PTI) [41,88]. The G2T39 genome also encoded acetolactate synthase, cystathionine beta-lyase, S-ribosylhomocysteine lyase, and alpha-acetolactate decarboxylase, which are involved in ISR in *B. velezensis* YC89 [15].

A number of putative genes related to plant growth-promoting functions metabolism were also identified in the G2T39 genome. A preliminary and presumptive nitrogen-fixing capacity was shown using Burk’s medium [24]. Further experiments using acetylene reduction or 15N incorporation should confirm these results. Moreover, putative genes involved in nitrogen metabolism in the G2T39 genome included a system comprising *nifS*, *nifU*, and *nifB* that encodes for nitrogenase. The *nifU* and *nifS* system has been characterized as very important for nitrogenase biosynthesis. Indeed, the *nifU* and *nifS* genes encode components of a cellular machinery dedicated to the assembly of [2Fe-2S] and [4Fe-4S] clusters required for growth under nitrogen-fixing conditions [47]. Moreover, NifB, which is the key protein involved in the synthesis of cofactors of all nitrogenases [46], was also found in the G2T39 genome. The genome also harbors the gene GlnK, which encodes protein PII that plays a key role in regulating nitrogenases [48,49]. Additionally, the gene *fixB*, which encodes flavoprotein involved in electron transfer for nitrogenase activation, as evidenced in *Azotobacter vinelandii* [45], was evidenced in G2T39. Moreover, the *narL/fixJ* system, which is involved in activating the expression of nitrogen fixation genes in response to low oxygen concentrations [89], was also found in the G2T39 genome. Recent studies have shown that *B. velezensis* strains are able to regulate nitrogen fixation [90,91]. However, characterization of nitrogenase-fixing systems in *B. velezensis* is still scarce, except that recent works pointed out the presence of *guaB* and *cysK*, *cysE*, and *ilvE* in *B. velezensis* strain CH1 [83]. The G2T39 strain is an endophytic bacteria isolated from the stem of a medicinal leguminous plant and, as such, does not possess nodulation genes and should not nodulate.

The G2T39 genome harbors a complex genetic system involved in phosphate metabolism, including a mixture of different pathways. Indeed, the G2T39 genome harbors a phytase homologous to PhyC isolated from *Bacillus subtilis* [55]. Phytases are enzymes involved in the catalysis of phytic acid hydrolysis by releasing phosphorus and consequently increasing its absorption by plants or animals [92]. Knowledge of Phyc functioning mechanisms is still scarce. However, the gene *pstA* in the genome of G2T39 may play a crucial role by allowing phosphate transport across the membrane, particularly in low-phosphate environments, which may therefore increase phytase activities. Indeed, the depletion of *phyC* and a *pst* gene was shown to increase the production of this phytase in *B. subtilis* BD170 [93]. Additionally, the G2T39 genome harbors the genes *glpQA*, *ugpE*, *ugpB*, *ugpC*, which are found in bacteria and encode glycerophosphoryl diester phosphodiesterases (GDPDs) involved in phospholipid hydrolysis. GDPDs have also been identified from archaea and bacteria [9,94,95]. These GDPDs have divergent pathways of functioning. For example, in *Escherichia coli*, UgpQ functions in the absence of other proteins encoded by the *ugp* operon and requires Mg^2+^, Mn^2+^, or Co^2^, in contrast to Ca^2+^-dependent periplasmic glycerophosphodiester phosphodiesterase GlpQ [95]. Additionally, *E. coli* uses a complex phosphonate pathway that involves not only the *uqp* gene clusters but also a *pst* cluster [56]. This is the first report of a complex phosphate metabolism system in an endophytic bacteria isolated from *C. retusa*, a medicinal plant. Since lipids constitute an important component of plant cells, GDPDs have been reported to be induced under Pi deficiency in several plant species [54,96,97]. However, the presence of this complex phosphate metabolism system in a plant endophytic bacteria may be very important for plant growth in phosphate-deficient environments.

## 5. Conclusions

This is the first report of the complete genome sequencing and analysis of an endophytic *B. velezensis* from the medicinal plant *C. retusa*. Our findings indicate that *B. velezensis* strain G2T39 could serve as a potential biocontrol agent to promote plant growth. This study demonstrated that G2T39 strain harbors multiple putative genes related to nitrogen metabolism/Fe–S cluster assembly and nitrogen regulation, phosphate metabolism, antifungal activity, plant resistance inducer biosynthesis, and various plant growth-promoting properties such as biofilm formation, siderophore biosynthesis, and zinc solubilization, making G2T39 a potential biocontrol agent and a biofertilizer.

## Figures and Tables

**Table 1:** Average nucleotide identity (ANI) values between *Bacillus velezensis* strain G2T39 and reference strains of *Bacillus* with available genome data.

	G2T39	
Strains	ANIb (%)	ANIm (%)
*B. velezensis* NRRL B-41580	98.83	99.00
*B. velezensis* KACC 18228	98.19	98.43
*B. velezensis* FZB42 ^1^	98.06	98.42
*B. velezensis* SQR9	97.99	98.32
*B. velezensis* KACC 13105	97.91	98.26
*B. amyloliquefaciens* XJ5	97.91	98.30
*B. ayatagriensis* RMG6	97.46	97.79
*B. siamensis* KCTC 13613	93.95	94.43
*B. siamensis* XY18	93.86	94.42
*B. amyloliquefaciens* DSM 7	93.55	94.26
*B. nakamurai* NRRL B-41091	85.65	87.08
*B. rugosus* SPB7	76.83	84.02
*B. mojavensis* KCTC 3706	76.34	84.00

^1^ *B. velezensis* strain FZB42 is a well-known Gram-positive *Bacillus* model strain for plant growth promotion and biocontrol studies [37].

**Table 2:** G2T39 genes as annotated in different databases.

Strain	Total CDS	VFDB	ARDB	CAZY	IPR	SWISSPROT	COG	CARD	GO	KEGG	NR	T3SS	Overall
G2T39	4118	187 (4.54%)	35 (0.84%)	207 (5.02%)	3444 (83.63%)	3323 (80.69%)	3030 (73.57%)	3 (0.07%)	2387 (57.96%)	2561 (62.19%)	4090 (99.32%)	417 (10.12%)	4099 (99.53%)

**Table 3:** Genome characteristics of *B. velezensis* G2T39 compared to reference strains XJ5 (CP071970.1), FZB42 (NC_009725), SQR9 (CP006890), and DSM7 (NC_014551.1).

Genome Characteristics	G2T39 Endophytic	XJ5 Plant	FZB42 Rhizosphere	SQR9 Rhizosphere	DSM 7 Soil
NCBI accession	CP199939	CP071970.1	NC_009725	CP006890	NC_014551.1
Size (base pairs)	4,040,830	4,160,003	3,918,596	4,117,023	3,980,199
CDS	3911	4307	3724	3957	4028
sORF	4	-	4	5	6
tRNA	87	87	90	72	94
rRNA	27	27	30	21	30
ncRNA	33	5	20	17	21
Prophages	4 (1, 1, 2)	4 (1, 3, 0)	3 (0, 3, 0)	5 (0, 3, 2)	8 (5, 3, 0)
Microsatellite/Minisatellite	0/36	0/17	1/31	0/20	2/74

**Table 4:** Secondary metabolites gene cluster types identified in the *Bacillus velezensis* G2T39 genome.

Region	Type	From	To	Similarity Confidence	Most Similar Known Cluster
1	NRPS	311,342	376,749	High	Surfactin
2	PKS-like	976,709	1,017,953	Low	-
3	Terpene	1,103,673	1,124,413	Low	-
4	TransAT-PKS	1,428,716	1,516,916	High	Macrolactin H
5	TransAT-PKS, T3PKS, NRPS-like, NRPS	1,741,965	1,852,076	High	Bacillaene
6	NRPS, betalactone	1,919,009	2,030,616	High	Fengycin
7	Terpene	2,066,805	2,088,688	Low	-
8	T3PKS	2,165,562	2,206,662	Low	-
9	TransAT-PKS	2,321,307	2,427,483	High	Difficidin
10	Terpene-precursor	2,450,817	2,471,707	Low	-
11	Terpene-precursor, NRP-metallophore, NRPS, RiPP-like	3,051,270	3,116,703	High	Bacillibactin
12	NRPS	3,415,561	3,483,981	High	
13	Other	3,686,785	3,728,203	High	Bacilysin
14	RiPP-like	3,905,907	3,918,985	Low	-

High: homology ≥ 75%; Low: homology < 50%.

**Table 5:** Comparison of CAZymes in the genomes of *Bacillus velezensis* G2T39 and related *Bacillus* FZB42 and SQR9.

*Bacillus* Strains	Glycoside Hydrolase Enzymes	Glycosyl Transferase Enzymes	Polysaccharide Lyase Enzymes	Carbohydrate Esterase Enzymes	Auxiliary Activity Enzymes	Carbohydrate-Binding Module Enzymes
G2T39	82	64	3	15	5	14
FZB42	38	40	3	14	5	12
SQR9	41	41	3	13	5	12

**Table 6:** Plant defense genes identified in the *Bacillus velezensis* G2T39 genome.

ID and Accession of Genes Identified in G2T39 Genome	Resistance Type	Gene Product	References
WP_038565169_1 *tuf*	PTI	Translation elongation factor EF-Tu	[39]
NP_390325_2 *efp*	PTI	Translation elongation factor EF-Tu	[40]
NP_389532_1 *tsf*	PTI	Translation elongation factor EF-Ts	[41]
NP_387891_1 *dacA*	PTI	D-alanyl-D-alanine carboxypeptidase	[15]
NP_390709_1 *ilvB*	ISR		[42]
NP_391482_2 *alsS*	ISR		[15,43]
WP_011197788_1 *metC*	ISR	Cystathionine beta-lyase/cystathionine gamma-synthase	[15,42]
NP_390945_1 *luxS*	ISR	S-ribosylhomocysteine lyase LuxS	[44]

**Table 7:** Nitrogen metabolism genes identified in the G2T39 genome.

ID and Accession of Genes Identified in G2T39 Genome	Gene Product	References
COG2025 *fixB*	Electron transfer flavoprotein, alpha subunit	[45]
COG1104 *nifS* COG1104 *nifS* COG1104 *nifS*	Cysteine desulfurase/Cysteine sulfinate desulfinase IscS or related enzyme, NifS family	[46] [47]
COG0347 *glnK*	Nitrogenase regulatory protein PII	[48] [49]
NP_388677_2 *skfB*	Putative Fe-S oxydoreductase	[46]
NP_391149_1 COG0719 *sufB*	Fe-S cluster assembly scaffold protein SufB	[50]
NP_391147_1 COG0822 *iscU*, *nifU*	Fe-S cluster assembly scaffold protein IscU, NifU family	[51]
NP_391096_1 *iscA*	Fe-S cluster assembly iron-binding protein IscA	[51]
*degU*	Activation of the nitrogenase	[52]

**Table 8:** Genes involved in phosphate metabolism.

ID and Accession of Genes Identified in G2T39 Genome	Gene Product	References
NP_388095_1 *glpQ*A	Glycerophosphoryl diester phosphodiesterase	[53] [54]
NP_389861_1 COG4247 *phyC*	3-phytase (myo-inositol-hexaphosphate 3-phosphohydrolase)	[55]
NP_387910_ *darA/pstA*	Cyclic di-AMP receptor DarA/PstA	[56]
COG0395 *ugpE* COG0395 *ugpE* COG0395 *ugpE* COG1175 *ugpA* COG1175 *ugpA* COG 1653 *ugpB* COG1653 *ugpB*	ABC-type glycerol-3-phosphate transport system, permease component	[56]

**Table 9:** Putative genes involved in other plant growth-promoting functions in the G2T39 genome. Predicted genes were selected when homology was superior to 80%.

ID and Accession of Genes Identified in G2T39 Genome	Gene Product	References	Function
NP_388218_1 *zinU*	Zinc metallochaperone YeiR/ZagA	[57]	Zinc solubilization
COG0307 *riB*	Riboflavin synthase alpha chain	[58]	Riboflavin synthesis
NP_390772_2 *yigB*	FMN and 5-amino-6-(5-phospho-D-ribitylamino)uracil phosphatase YigB, HAD superfamily	[59]	Riboflavin synthesis
NP_390206_1 *ribH*	6,7-dimethyl-8-ribityllumazine synthase	[58] [59]	Riboflavin synthesis
NP_388042_2 *feuC* NP_388043_1 *feuB* NP_391211_1 *fhuG* NP_391173_1 feuV NP_391210_1 *fhuC*	ABC-type Fe3+-siderophore transport system, permease component	[60] [61] [62] [63] [64]	Siderophore transport and metabolism
NP_387913_1 *ricT*	Cell fate regulator YaaT, PSP1 superfamily (controls sporulation, competence, biofilm development)	[65]	Biofilm formation
COG3679 *ylbF* COG3679 *ylbF*	Cell fate regulator YlbF, YheA/YmcA/DUF963 family (controls sporulation, competence, biofilm development)	[66]	Biofilm formation
NP_390738_1 *yshB*	Colicin V production accessory protein CvpA, regulator of purF expression and biofilm formation	[67]	Biofilm formation
NP_388171_1yceC NP_388173_1 *yceE*	Stress response protein SCP2	Putative [41]	Stress tolerance
NP_390361_2	Thiamin-binding stress response protein YqgV, UPF0045 family	Putative [41]	Stress tolerance
NP_391606_1 *narL* NP_391607_1 *narJ* NP_391608_1 *narH* NP_391609_2 *narG*	Nitrate reductase assembly protein NarJ, required for insertion of molybdenum cofactor	[68]	Nitrate reductase
NP_391284_1 *lutB*	L-lactate utilization protein LutB, contains a ferredoxin-type domain	[69]	Lactate metabolism
COG1652 kbp	Cytoplasmic potassium-binding protein Kbp/XkdP/YgaU, contains LysM domain	[70]	Potassium homeostasis
COG1333 *resB*	Cytochrome c biogenesis protein ResB	[71]	Cytochrome c biogenesis
NP_832865_1 NP_387898_1 NP_391054_1	Nicotinamidase-related amidase	[72]	Nicotinamide Biosynthesis

## Data Availability

The genome sequence of the isolate G2T39 was deposited at NCBI under the GenBank accession number CP199939.

## Supplementary Materials

The following supporting information can be downloaded at: https://www.mdpi.com/article/10.3390/microorganisms14010123/s1, Figure S1: Distribution of genes across KEGG functional categories in G2T39 genome; Figure S2: Distribution of genes across GO functional categories in the G2T39 genome; Figure S3: Growth of strain G2T39 on Burk’s medium; Table S1: Inhibition rate of *B. velezensis* strain G2T39 on pathogenic fungi; Table S2: List of annotated CAZymes in the genome of G2T39; Table S3: relative abundance of different CAZymes classes in the genome of G2T39.
