# Supplementary material for: Whole-Genome Sequencing and Analysis Reveals Plant Growth-Promoting Properties and Biocontrol Potential of the Crotalaria retusa Endophytic Bacillus velezensis Strain G2T39
Source: Microorganisms. 2026 Jan 7;14(1):123. doi: 10.3390/microorganisms14010123 (PMC12843875; doi:10.3390/microorganisms14010123)
Supplement: Supplementary file 1 [file microorganisms-14-00123-s001.zip › Supplementary figure.pdf]

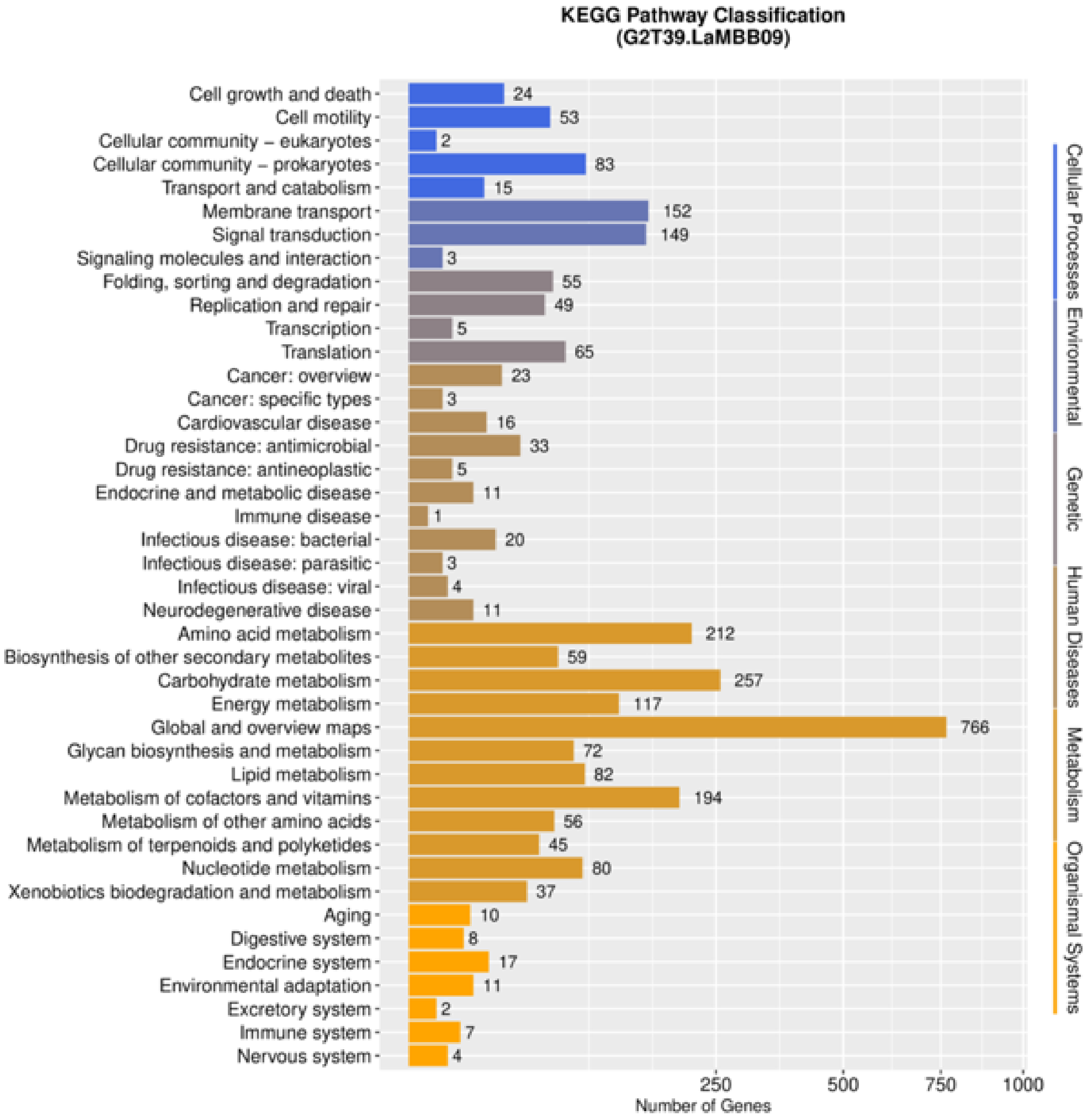

Figure S1: Distribution of genes across KEGG functional categories in G2T39 genome

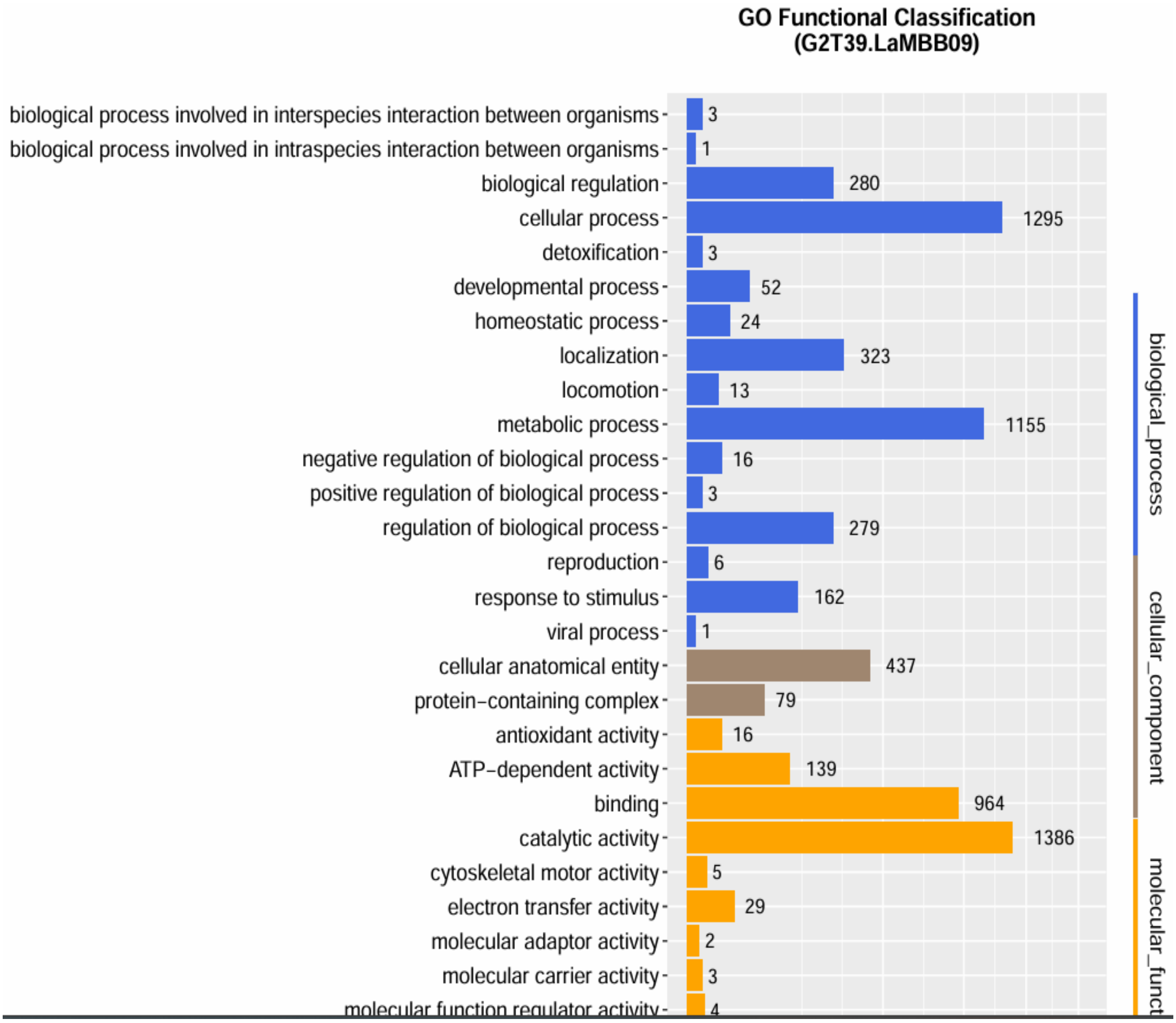

Figure S2: Distribution of genes across GO functional categories in the G2T39 genome

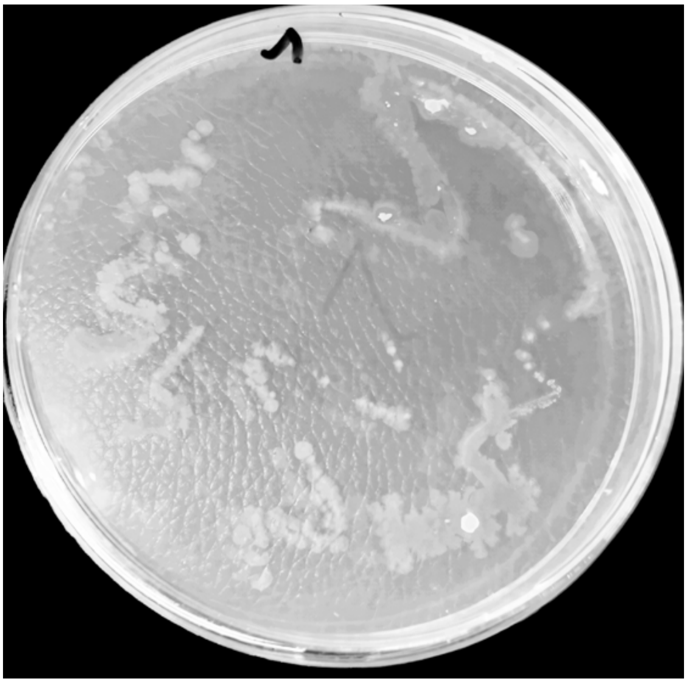

Figure S3: Growth of strain G2T39 on Burk’s medium
