# Supplementary material for: Whole-Genome Sequencing and Analysis Reveals Plant Growth-Promoting Properties and Biocontrol Potential of the Crotalaria retusa Endophytic Bacillus velezensis Strain G2T39
Source: Microorganisms. 2026 Jan 7;14(1):123. doi: 10.3390/microorganisms14010123 (PMC12843875; doi:10.3390/microorganisms14010123)
Supplement: Supplementary file 1 [file microorganisms-14-00123-s001.zip › Supplementary table S1.pdf]

Table S1: Inhibition rate of *B. velezensis* strain G2T39 on pathogenic fungi

Pathogen diameter Dassay (cm)						
Phytopathogen	Assay1	Assay 2	Assay 3	Means	Diameter of control Dcontrol (cm)	Inhibition rate (%)
Colletotrichum gloeosporioides	3.7	4.8	5	4.5	6.4	0.296875
Fusarium oxysporum f. sp. *vasinfectum* (FOV)	3.3	3.4	3.5	3.4	6.2	0.451613

Inhibition rate = 100 * (1 - Dassay/Dcontrol)
